# Genome-wide DNA methylation profiling in anorexia nervosa discordant identical twins

**DOI:** 10.1038/s41398-021-01776-y

**Published:** 2022-01-10

**Authors:** C. Iranzo-Tatay, D. Hervas-Marin, L. M. Rojo-Bofill, D. Garcia, F. J. Vaz-Leal, I. Calabria, L. Beato-Fernandez, S. Oltra, J. Sandoval, L. Rojo-Moreno

**Affiliations:** 1 Psychiatry Service, Hospital la Fe, Valencia, Spain; 2 Department of Psychiatry, Medicine School, University of Valencia, Valencia, Spain; 3 Department of Applied Statistics and Operational Research and Quality, Universitat Politècnica de València, Valencia, Spain; 4 Epigenomics Unit, Instituto de Investigación Sanitaria La Fe, Valencia, Spain; 5 Department of Psychiatry, Medicine School, University of Extremadura, Badajoz, Spain; 6 Eating Disorders and Children’s Psychiatry Department, Hospital General, Ciudad Real, Spain; 7 Genetics and Prenatal Diagnosis Unit, Hospital La fe, Valencia, Spain; 8 Biomarkers and Precision Medicine Unit (UByMP), Instituto de Investigación Sanitaria La Fe, Valencia, Spain; 9 Consorcio de Investigación Biomédica en Red de Epidemiología y Salud Pública (CIBERESP), Madrid, Spain

**Keywords:** Psychiatric disorders, Clinical genetics

## Abstract

Up until now, no study has looked specifically at epigenomic landscapes throughout twin samples, discordant for Anorexia nervosa (AN). Our goal was to find evidence to confirm the hypothesis that epigenetic variations play a key role in the aetiology of AN. In this study, we quantified genome-wide patterns of DNA methylation using the Infinium Human DNA Methylation EPIC BeadChip array (“850 K”) in DNA samples isolated from whole blood collected from a group of 7 monozygotic twin pairs discordant for AN. Results were then validated performing a genome-wide DNA methylation profiling using DNA extracted from whole blood of a group of non-family-related AN patients and a group of healthy controls. Our first analysis using the twin sample revealed 9 CpGs associated to a gene. The validation analysis showed two statistically significant CpGs with the rank regression method related to two genes associated to metabolic traits, PPP2R2C and CHST1. When doing beta regression, 6 of them showed statistically significant differences, including 3 CpGs associated to genes JAM3, UBAP2L and SYNJ2. Finally, the overall pattern of results shows genetic links to phenotypes which the literature has constantly related to AN, including metabolic and psychological traits. The genes PPP2R2C and CHST1 have both been linked to the metabolic traits type 2 diabetes through GWAS studies. The genes UBAP2L and SYNJ2 have been related to other psychiatric comorbidity.

## Introduction

Eating disorders (EDs) are one of the major psychiatric conditions, being anorexia nervosa (AN) one of the illness with the highest mortality among these maladies [1]. AN constitutes a serious emerging health problem, affecting mainly adolescents in developed countries. AN’s diagnosis is established based on purely clinical criteria, given that there are no biological, genetic and epigenetic markers.

Mental disorders are known to be complex conditions in which many environmental and genetic risk factors interact. The role of genetics factors was initially proven via family, twin and adoption investigations [2]. By these means, moderate-to-high heritability estimates have been demonstrated in mental disorders [3], up to 64% in schizophrenia, over 70% in bipolar disorder [4], or a range of 22% to over 62% in ED [5], among others. Furthermore, molecular genetic investigations have pictured all these psychiatric conditions as highly polygenic [6]. However, we cannot fail to forget the main role that the environmental factors play in the mental disorders’ aetiology. Research has continuously demonstrated that the environment may influence the development of these disorders. Numerous environmental factors have been epidemiologically associated with psychiatric maladies, comprising psychosocial adversities, parent’s mental health, violence, stress or the intrauterine exposure to tobacco smoke and alcohol, among others [7].

There is a substantial amount of evidence to fully support that the roles of genetic and environmental factors depend on each other [3]. In this regard, multiple lines of evidence suggest that the development and maintenance of mental disorders is due to gene–environment interactions (GxE) that alter genetic expression via epigenetic processes [8]. These processes may explain the inconsistency between the high inheritance of psychiatric disorders and the lack of strong related genetic markers [9]. Epigenetic changes may regulate gene transcription and function independently of the DNA sequence [10–12]. Epigenetic mechanisms include DNA methylation, post-translational histone modifications, higher order chromatin remodelling, and non-coding RNA (ncRNA) regulation. DNA methylation is widely hypothesised to be a mechanism by which both heritable and environmental factors can influence the regulation of gene expression and function [13]. The most widely studied epigenetic change in humans is DNA methylation of cytosines at CpG dinucleotides [11].

Regarding EDs, the number of epigenetic studies which have been conducted in this field are limited. These overall investigations have been developed until today by means of clinical samples, rather than specifically twin samples. To date, as far as we are aware, just one study has looked specifically at epigenetic changes in AN throughout discordant twin samples [14], but their study design was different to ours, by using twin sample collection just as validating cohort. Identical twins can be considered replicates of the same genomic sequence. Thus, investigating discordant MZ twin pairs is a powerful strategy for uncovering disease-associated epigenetic changes because it allows an assessment of the epigenome independent of any underlying genomic sequence variation [13, 15]. Epigenetic differences between MZ twins accumulate with age. This epigenetic drift may explain why monozygotic twins are often discordant for EDs [10]. Hence, using identical twins for epigenetic studies is the most efficient design available as it controls for genetic factors, age, cohort effects and many environmental influences that otherwise add variability and noise [16].

The aim of the study was to discover the epigenetic bases of AN. In order to do so, we quantified genome-wide patterns of DNA methylation using the Illumina Infinium HumanDNAMethylation 850 BeadChip (“850 K array”) in DNA samples isolated from whole blood collected from a group of discordant AN twins. We then validated the results with genome-wide DNA methylation profiling using a group of not family-related AN patients and a group of healthy controls.

## Material and methods

### Study design

This study was approved by the Ethics Committee (Biomedical Investigation Ethics Committee of La Fe University Hospital of Valencia, Spain) and was conducted in accordance with the guidelines of the Declaration of Helsinki. Signed informed consent was obtained from each participant in the study or participants’ relative in the case of underaged participants.

Patients were recruited as part of both discovery and validation cohorts. The Discovery cohort was composed by Monozygotic twin pairs, discordant for AN, recruited through the study “Twin and family anorexia nervosa patients’ registry” which has been run at the Eating Disorders Unit of La Fe Hospital (Valencia, Spain) since 2011. This project was originally based on blood donation samples from patients and their first-degree relatives (parents and siblings) to the Biobank of the Public Health Investigation Foundation of the Community of Valencia (IBSP-CV). Patients who voluntarily consented to participate in the research and donated a blood sample (prior verbal and written information), were recruited at La Fe Hospital at the ED inpatient unit, ED outpatient unit, or the ED day hospital. The validation cohort consisted of unrelated AN case that was also recruited from the different ED care units of La Fe Hospital. Their blood sample was processed and stored by the Biobank of La Fe Hospital. Finally, control group comprised 7 NED women (medicine students and medical doctors) matched by clinical characteristics. None of the women in the NED group used psychoactive medications. Case definition required a lifetime diagnosis of AN (restricting or binge-purge subtype) following DSM-IV-TR diagnostic criteria. Given that amenorrhea does not increase diagnostic specificity (and the DSM-5 has removed it as a diagnostic criterion), its presence was not required.

All participants underwent a physical evaluation including age, toxic habits, and measures of weight, height, body mass index (BMI) and nutritional status. Moreover, sociodemographic variables were also obtained.

Twin blood samples were processed, isolating plasma, and conserved at −80 °C by the Biobank of the Public Health Investigation Foundation of the Community of Valencia (IBSP-CV). Unrelated AN cases and NED blood samples were processed and stored by the Biobank of La Fe Hospital.

Genomic DNA (gDNA) from plasma was extracted using the QIAcube from Qiagen® and the QIAamp® DNA Investigator Kit (Qiagen®, Germany), according to the manufacturer’s protocol. Prior the methylation studies, a DNA integrity quality control was performed to ensure that the DNA met the standard quality measurements that included a minimum requirement of 200 ng. All DNA samples were treated with RNaseA for 1 h at 45 °C, quantified by the fluorometric method (Quant-iT PicoGreen dsDNA Assay, Life Technologies, CA, USA), and assessed for purity by NanoDrop (Thermo Scientific, MA, USA) 260/280 and 260/230 ratio measurements. DNA integrity of the fresh frozen samples was checked by electrophoresis on a 1.3% agarose gel.

### Zygosity typing of cases

The Mentype® Chimera® Kit (Biotype) was used following the manufacturer’s instructions. Genetic markers are distributed over 12 chromosomes and represent highly polymorphic short tandem repeats (STRs) with a very high rate of heterozygosity and a balanced allelic distribution (loci *D2S1360, D3S1744, D4S2366, D5S2500, D6S474, D7S1517, D8S1132, D10S2325, D12S391, D18S51, D21S2055, SE33-ACTBP2*) and the gender-specific locus Amelogenin. The amplified products were determined by capillary electrophoresis on an ABI Prism 3130XL automated sequencer (Applied Biosystems).

### Genome-wide DNA methylation analyses

The epigenomic study was performed using the Human DNA Methylation EPIC BeadChip array that interrogates around 850,000 CpGs as previously described (Moran et al., 2016). This technology is highly reliable in detecting epigenetic alterations (Lorente-Pozo et al.). All purified DNA samples were randomly distributed into 96‐well plates. Bisulfite conversion of 600 ng of genomic DNA was performed using the EZ‐96 DNA Methylation Kit (Zymo Research Corp., Irvine, CA, USA) following the manufacturer’s recommendations. About 200 ng of bisulfite‐converted DNA was used for hybridisation on the Infinium DNA MethylationEPIC BeadChip (Illumina). Briefly, samples were whole‐genome‐amplified followed by an enzymatic endpoint fragmentation, precipitation and resuspension. The resuspended samples were hybridised onto the BeadChip for 16 h at 48 °C and washed. A single‐nucleotide extension with labelled dideoxynucleotides was performed, and repeated rounds of staining were applied with a combination of labelled antibodies differentiating between biotin and 2,4‐dinitrophenol (DNP).

### Data normalisation

The methylation score for each CpG was represented as a beta value according to the fluorescent intensity ratio. Beta values may take any value between 0 (no methylated) and 1 (completely methylated). The raw data (IDAT files) were normalised using functional normalisation as implemented in the R-package minfi (version 1.22.1). CpG markers present on Methylation EPIC are classified based on its chromosome location and the feature category gene region as per UCSC annotation (TSS200, TSS1500, 5′UTR, 1st Exon, Body, 3′UTR). This array has a complete coverage of the genome including 99% of the genes described. Additional criteria included the location of the marker relative to the CpG substructure (island, shore, shelf, open sea), fantom 5-associated enhancer regions and regulatory regions described on ENCODE project, such as transcription binding site sequences, open chromatin regions and DNase I hypersensitivity clusters.

Every beta value in the EPIC array is accompanied by a detection *p*-value which represents the confidence of a given beta value. Probes and sample filtering involved a two-step process in which unreliable betas with high detection *P* value *P* > 0.01 (1712 CpGs), 2931 CpGs associated with SNPs and cross-hybridising probes identified in the were removed. Previous analyses have indicated that a threshold value of 0.01 allows a clear distinction to be made between reliable and unreliable beta values [17]. After the filtering, the remaining 861254 CpGs were considered valid for the study. The omic data (raw, processed and metadata) is accessible in the GEO repository with the following code: GSE186766.

### Statistical analyses

An exploratory analysis was first performed on the twin dataset using PCA and a heatmap with a random sample of 5000 CpGs. For the differential methylation analysis, a mixed-effects rank-based regression model was adjusted. The non-independent nature of the paired samples was considered by the model by including a random intercept for each pair of twins. The resulting *p*-values were adjusted for multiple comparisons using False Discovery Rate (FDR). After the FDR corrections, a list of differentially methylated CpGs was obtained for FDR-adjusted *p*-values < 0.05. This list of CpGs was then validated on the general population cohort. The validation was performed by adjusting a rank-based model for each of the differentially methylated CpGs. An additional sensitivity analysis was performed by adjusting beta regression instead of rank-based models. All statistical analyses were performed using *R* (version 4.0.1) and R packages *Rfit* (version 0.23.0), *betareg* (version 3.1-1), *IlluminaHumanMethylation450kmanifest* (version 0.4.0) and *pcaMethods* (version 1.74.0).

## Results

### Clinical characteristics

The clinical characteristics of the patients and donors from both cohorts are shown in Table 1. Groups did not vary according to mean age but did differ as expected in terms of mean BMI. Regarding smoking habits, only two twin pairs were smokers, one of the twin pairs were social smokers (smoked 1–2 cigarettes per day), and the other twin pair couple were habitual smoker (smoked >10 cigarettes per day).

**Table 1:** Clinical characteristics of the samples from both cohorts, The twin sample was composed of 7 MZ twin pairs. The validating sample was composed of 7 matched controls by age and BMI, and 7 matched AN patients by age and BMI.

Sample	Age (sd)	BMI (sd)
MZ AN	20,42 (7,39)	16,75 (1,43)
MZ control	20,42 (7,39)	19,85 (1,06)
AN	20,14 (7,08)	15,62 (0,95)
Control	21,42 (7,48)	19,87 (1,2)

*AN* anorexia nervosa, *BMI* body mass index, *MZ* monozigotyc, *sd* standard deviation.

Our AN MZ twin sample was composited with 7 women between 14 and 35 years old, and their BMI varied between 15 and 19. The fact that our AN twin sample showed a mean BMI of 16,75 (1,43 st) emphasises the fact that our potential findings might be related to other different traits of the illness rather than to solely weight issues. DSM V does not establish a specific weight that the patient must meet to concurrent the criteria to be diagnosed with AN. The DSM 5 AN criteria go as follows; (a) restriction of food intake leading to weight loss or failure to gain weight resulting in a “significantly low body weight” of what should be expected for someone’s age, sex and height. (b) fear of becoming fat or gaining weight. (c) Have distorted view of themselves and of their condition.

### Zygosity typing of cases

**Fig. 1:**
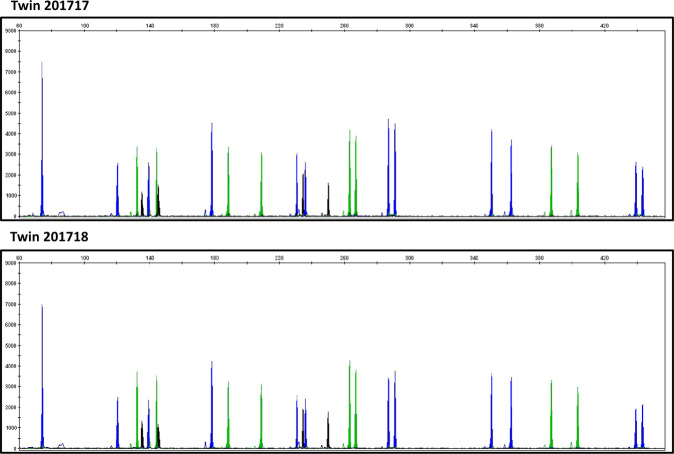
Monozigosity analysis in the discovery cohort. Two representative examples of the determination of monozygosity using microsatellite markers.

In all cases, the monozygotic twin status was confirmed by microsatellite analysis of the DNA obtained from peripheral lymphocytes (Fig. 1).

### Identification of novel epigenetic loci associated to AN

**Fig. 2:**
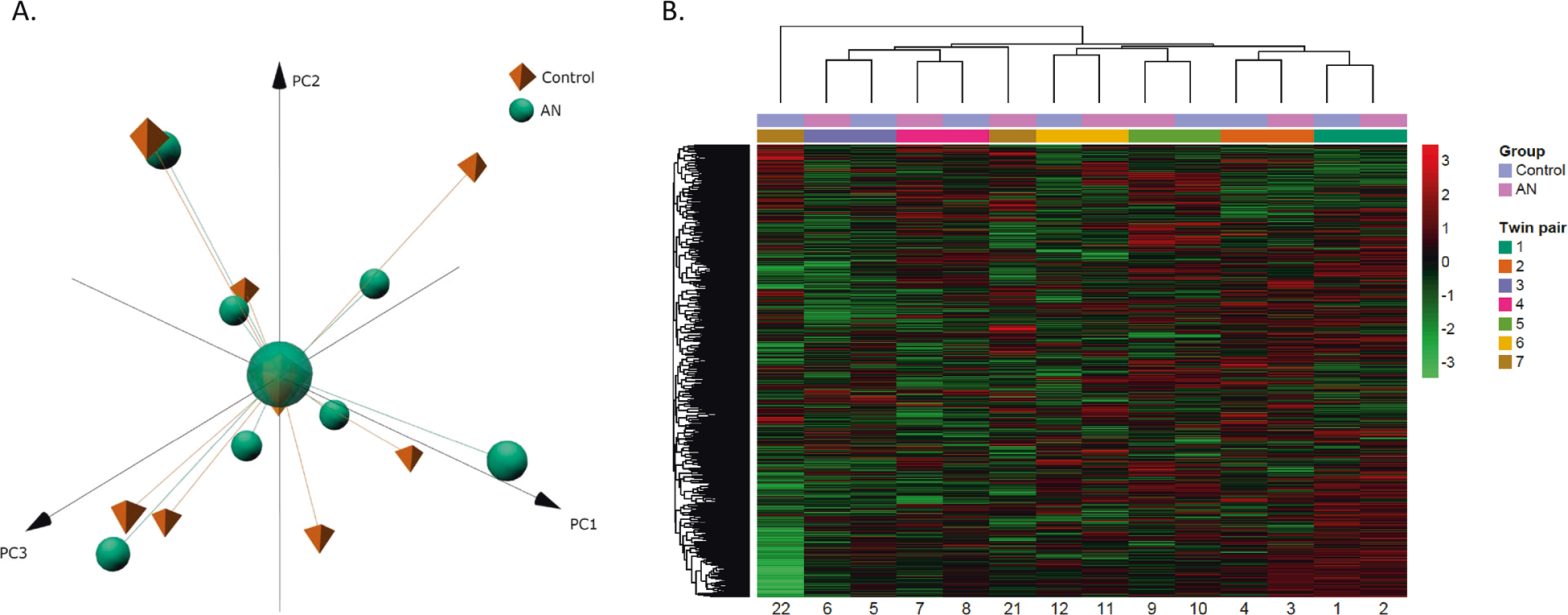
Analysis of the global DNA methylation profile in the twin cohort. **A** Representation of the Principal Component Analysis (PCA) on the methylation data. Centroids of each group of patients are represented by different shapes and colours. **B** Heatmap with a random sample of 5000 CpGs from the twin cohort. *Z*-score colour scale ranges from green for lower methylation to red for higher methylation levels.

First, an exploratory analysis was performed by running a principal component analysis (PCA) and a heatmap with hierarchical clustering. This analysis was used to visualise overall differences among groups, similarities between twins and also to detect potential outliers. Results revealed no evident differences between both groups (Fig. 2A, B). To better visualisation, we also depict two dimensions plots (Supplementary Fig. 1). Regarding twin pairs, all of them except one were very similar, pairing together in both the PCA analysis and the heatmap (Supplementary Figs. 2 and 3).

**Fig. 3:**
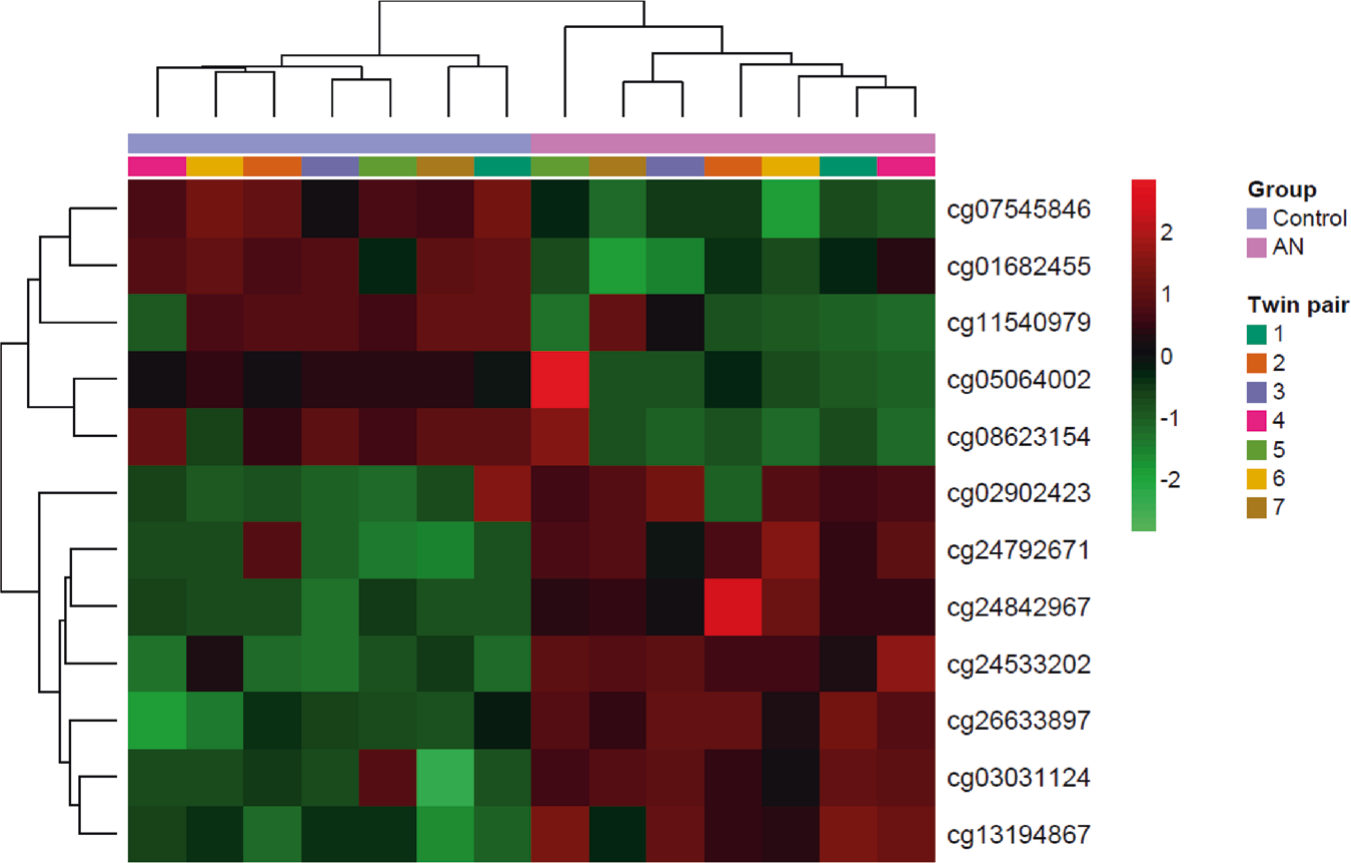
Identification of a DNA-methylation signature characterising the differences between AN and control twins. Heatmap with the methylation status of the differentiating CpGs between AN and control twins. Rows (CpGs) and columns (individuals) are ordered according to the results of a hierarchical clustering algorithm. *Z*-score colour scale ranges from green for lower methylation to red for higher methylation levels.

In order to identify differentially methylated CpGs characterising AN, we performed a differential methylation analysis adjusting rank-based mixed regression models to each CpGs. After correction for multiple comparisons with FDR, 12 CpG remained statistically significant. Of these, 9 CpGs were associated to a gene. These genes were; at chromosome (Cr) 1 genes *UBAP2L* and *LMNA*, Cr 4 *PPP2R2C*, Cr 6 *SYNJ2*, Cr 9 gene *ZER1*, at Cr 11 genes *CHST1* and *JAM3*, at Cr 12 *TUBA1A* and at Cr 19 gene *FCHO1*. The results of this supervised analysis are depicted in a clustered heatmap (Fig. 3) and presented in Table 2. Additionally, in supplementary table 1, we provide CpG features from Illumina manifest.

**Table 2:** Identification of novel epigenetic loci associated to AN and results of the exploratory analysis in the validation cohort.

IlmnID	UCSC_RefGene_Name	Adjusted *p*-value	*p*-value validation cohort (rank based)	*p*-value validation cohort (beta regression)
cg05064002	FCHO1	1.58E-05	0.48	0.19
cg13194867	–	0.005	0.087	0.003
cg07545846	JAM3	0.041	0.16	0.024
cg03031124	ZER1	0.008	0.66	0.89
cg24842967	LMNA	4.35E-05	0.37	0.47
cg08623154	UBAP2L	1.30E-04	0.15	0.017
cg02902423	PPP2R2C	0.038	0.039	0.005
cg24792671	–	0.026	0.43	0.28
cg26633897	–	5.10E-04	0.57	0.61
cg11540979	SYNJ2	0.025	0.086	0.01
cg01682455	CHST1	0.034	0.037	0.03
cg24533202	TUBA1A	0.038	0.88	0.92

*IlmnID* Illumina identification, *UCSC* University of California Santa Cruz.

### Validation in a new unrelated cohort

Once we had identified the differentially methylated CpGs in our initial discovery twin cohort, we sought to compare the global DNA methylation profile and to validate these previously identified single DNA methylation markers in an additional unrelated cohort. Therefore, we performed the Infinium DNA Methylation EPIC array of unrelated 7 AN patients and 7 NED donors. Clinical characteristics are depicted in Table 1.

**Fig. 4:**
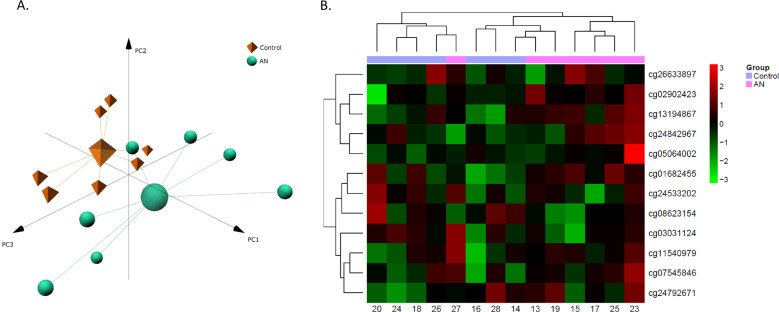
Validation of the DNA methylation signature in the non-twin validation cohort. **A** Representation of the Principal Component Analysis (PCA) on the methylation data. Centroids of each group of patients are represented by different shapes and colours. **B** Heatmap with the methylation status of the differentiating CpGs between AN and control individuals from the non-twin cohort. Rows (CpGs) and columns (individuals) are ordered according to the results of a hierarchical clustering algorithm. *Z*-score colour scale ranges from green for lower methylation to red for higher methylation levels.

**Fig. 5:**
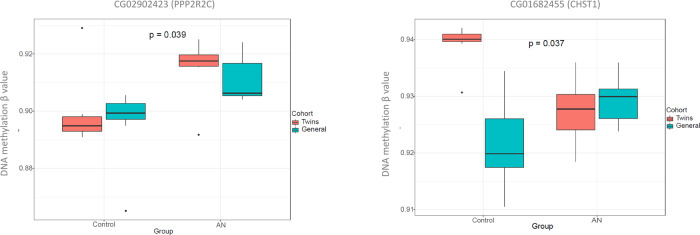
DNA methylation levels for the validated CpGs between AN and controls in both cohorts. Box plots of the validated CpGs associated with the PPP2R2C and CHST1 depicting differences in methylation levels between AN and controls in the twin cohort (red) and in the non-twin cohort (blue).

As expected, due to the variability in unrelated human samples, results of the exploratory analysis in the validation cohort were slightly different to those in the twins cohort, finding subtle differences between both groups in the global methylation pattern, which was only evident in the PCA analysis but not in the clustered heatmap (Fig. 4A, B). For the validation, only the CpGs selected from the first cohort were analyzed. Of these, half of them showed the same effect and similar effect size in the validation cohort compared to the discovery cohort, but only two of them, cg02902423 (*PPP2R2C*) and cg01682455 (*CHST1*), were statistically significant with the rank regression method (Fig. 5). Since rank regression methods may suffer from a lack of statistical power for being non-parametric, we additionally performed a beta regression analysis which, as a parametric method, maximises power when assumptions are met. With this second analysis, 6 of them showed statistically significant differences: both previously described and cg13194867 (intergenic region), cg07545846 (*JAM3*), cg08623154 (*UBAP2L*) and cg11540979 (*SYNJ2*) (Supplementary Fig. 4). Results of the validation cohort are also described in Table 2.

## Discussion

Previous research concerning epigenetic changes in EDs have been conducted majorly in non-twin samples. In this regard, Frieling et al. observed elevated methylation levels at the promoter region of the atrial natriuretic peptide in patients with Bulimia Nervosa (BN) [18], hypermethylation of the dopamine transporter (DAT) promoter region in BN, and hypermethylation of the dopamine D2 receptor (DRD2) promoter in AN patients [19]. Furthermore, Groleau and Steiger, associated methylation changes at the DRD2 promoter region, and the promoter gen of the BDNF with comorbid psychopathology in patients with ED [20]. They also described that methylation levels of the glucocorticoid receptor gene (NR3C1) promoter are higher in women with BN and comorbid borderline personality disorder than in women with BN and no comorbidity [21]. In addition, the research group at the University of Seoul, has described higher levels of methylation of the oxytocin receptor gene in AN [22]. Moreover, decreased global DNA methylation levels have been described in patients suffering from AN, by the research team of the University of Milan [23]. On the contrary, neither the research team of Saffrey and colleagues nor the research group of Pjetri *et al*, have been able to detect methylation differences in AN when compared to controls [24, 25]. Extended information regarding epigenetics on EDs is detailed in specific reviews [8] and systematic reviews papers [26, 27].

A recent work carried out by Steiger et al. [28] showed new interesting insights in the epigenomic field of EDs. They measured genome-wide DNA methylation in 75 women with active AN, 31 women who had previously fulfilled the DSM­5 criteria for AN but not upon entry into the study and 41 non­eating­disordered (NED) women. Their findings described 58 differentially methylated sites related to genes relevant to metabolic and nutritional status (lipid and glucose metabolism), psychiatric status (serotonin receptor activity) and immune function. Even though their discoveries were remarkable, some limitations should be considered. Analyses of the majority of the sample were conducted using the previous Infinium Human Methylation 450 BeadChip Kit (Illumina Inc.; *n* = 171 samples), while the Infinium DNA MethylationEPIC BeadChip Kit which provides much more information, was only implemented in a limited sample size (Illumina Inc.; *n* = 28 samples). Furthermore, their model did not include a twin sample discordant for the condition under study.

Finally, Kesselmeier et al. analysed high-throughput DNA methylation data derived from whole blood for differences between 47 AN patients and two control groups; 47 lean (BMI ≤ 15) females and population-based female controls. They then used a validation sample of 5 monozygotic AN-discordant twin pairs. They detected that multiple CpG sites at the TNXB gene were hypermethylated in their AN patients. Mutations of the TXNB gene have been related to the Ehlers-Danlos syndrome, macular degeneration and vesicouretral refloux [14].

Our investigation is the first comprehensive examination of disorder-associated DNA methylation differences in MZ twins discordant for AN, by means of a genome-wide approach, validating our findings in an unrelated sample of AN patients and controls.

Different methylation patterns in several specific genomic regions were observed in patients compared with controls. The validation analysis showed two statistically significant CpGs with the rank regression method related to two genes associated to metabolic traits, *PPP2R2C* and *CHST1*, which have been linked to the metabolic trait type 2 diabetes.

Type 2 diabetes and abnormalities in insulin secretion and glucose levels have been linked to AN. The long-term nutritional deprivation that occurs in AN leads to a state of acquired Growth Hormone (GH) resistance that results in an increased GH secretion but decreased systemic insulin-like growth factor 1 (IGF1) and low levels of insulin [29]. It has been suggested that AN shares genetic variation with various metabolic phenotypes, including fasting insulin, leptin, insulin resistance, type 2 diabetes, and HDL cholesterol, which may be independent of BMI [30]. In line with our research, a SNP variant of the *PPP2R2C* gene (rs4689388) at the alleles G/A has been associated to type 2 diabetes [31]. Additionally, the *PPP2R2C* gen has been mapped to weight traits, such as BMI and body weight [32] by means of GWAS investigations. Also, the *CHST1* gene was associated with type 2 diabetes at a meta-analysis of GWAS studies conducted by Wood et al. [33], but did not reach statistical significance.

Moreover, some of the genes that showed different methylation patterns have been related in some way to body weight and other metabolic traits. In this regard, the *FCHO1* is a protein-coding gene that regulates Bone Morphogenetic Proteins (BMPs) signalling by regulating clathrin-mediated endocytosis of BMPs receptors. The BMPs family plays a role in multiple metabolic and reproductive pathways [34]. The BMPs family, has been related to obesity [35], and, more concretely, the BMP receptor 1A gene is an important regulator of adiposity. Furthermore, Winkler et al. [36] performed meta-analyses that included 320,485 adults of European ancestry from 114 studies with genome-wide array and in which a SNP variant (rs2270204) of the *ZER1* gene at the alleles T/G was related with the trait BMI. Finally, a recent GWAS study described that the *LMNA* gene is related to birth weight [37]. Birth weight has been observationally associated with future risk of adult cardiometabolic diseases in the offspring, such as diabetes. In fact, a study run by Duncan et al. identified, by means of a GWAS on a sample of 3495 AN cases one genome-wide significant locus on chromosome 12 (rs4622308) in a region harbouring a previously reported type 1 diabetes and rheumatoid arthritis locus [38]. Regarding other metabolic traits, our work pointed towards two genes that GWAS studies have described to be involved in the expression of the high-density lipoprotein (HDL), *TUBA1A* [39] located in Cr 12 and *SYNJ2* [40] located in Cr 6.

The BMPs family whose signalling, as stated before, may be regulated by the *FCHO1* gene, has been related to reproductive pathways [34]. Animal models have demonstrated that fertility is influenced by several BMPs. For instance, *BMP15* knockout female mice are subfertile and have lower ovulation and fertilisation rates [41] and so are *BMP6* knockout female mice, with a decrease in ovulated eggs [42]. Furthermore, other BMPs are required for an adequate post-implantation uterine function and pregnancy maintenance [43]. Therefore, our findings may be linked to the lifetime fertility issues experienced by some patients with an ED.

Interestingly, the gene *ZER1* found in our research was previously targeted by the group of Wade et al, who found out that a single nucleotide polimorfism variant (rs514024) of the gene at the alleles G/A was associated to EDs. More precisely, it was linked to the phenotype “purging via substances” [44]. These authors did not evaluate methylation levels, their twin sample was not discordant for the pathology and no variants reached genome-wide significance at the level of *p* < 10 − 8. However, their findings are specially relevant, and, specially, the possibility that abnormalities in this gene, which encodes a subunit of an E3 ubiquitin ligase complex that may be involved in meiosis, could be related to EDs through purging behaviours such as starvation, excessive exercise, laxatives, fluid tablets, slimming tablets or self-induced vomiting [44].

Lastly, our findings regarding the association of EDs with other psychopathology should also be highlighted. As known, psychiatric comorbidities are very common in AN and include conditions, like mood and anxiety disorders, obsessive-compulsive disorders, personality disorders and substance abuse traits [45]. Shared genetic factors may trigger the coexpression of these comorbidities. Two SNP of our list of genes were related to psychiatric comorbidity by means of GWAS studies. *UBAP2L* (Ubiquitin Associated Protein 2 Like) is a protein-coding gene that plays an important role in the activity of long-term repopulating hematopoietic stem cells [46]. A SNP variant of the *UBAPL2* gene (rs115462819) at the alleles C/T has been associated with the phenotype Bipolar disorder via a GWAS investigation, not reaching statistical significance [47]. Furthermore, the *SYNJ2* gene, member of the inositol-polyphosphate 5-phosphatase family, has been associated with cannabis use initiation but, again, without reaching genome-wide significance [48]. It is worth noting that the endocannabinoid system has been associated with diverse physiological functions, including the regulation of appetite, food intake and energy balance, and has a key role in brain reward systems and in maintaining psychophysiological homeostasis [49]. On other hand, Watson et al., in their paper found evidence implying a metabo-psychiatric origin for anorexia nervosa. Interestingly, in our paper we find alteration in genes related to other psychiatric morbidities such as UBAP2L and SYNJ2 and find also alteration in metabolic/diabetogenic genes such as PPP2R2C and CHST1. Although these genes are not reported in Watson’s study, they also point to genes related to psychiatric disorders and the glycemic metabolism. Furthermore, it has been usually stated that genetics and epigenetics are mutually exclusive mechanisms for gene regulation. So, our study supports the conclusions of Watson et al. [30] paper, further providing arguments for the reconceptualization of anorexia nervosa as a metabo-psychiatric disorder.

The overall pattern of results from the genome-wide methylation analysis of AN shows genetic links to interesting phenotypes that the literature has constantly related to AN, including metabolic and psychological traits. More important than the significance threshold in any study is the accumulation of evidence that genes, and, in particular, specific loci contribute to phenotypic variation. Further studies are planned to be carried out in the future to gain knowledge in this unexplored aspect of the disease. Whole-genome deep sequencing, using the same samples from both cohort of the study, will provide a comprehensive characterisation of the genetic features, including postzygotic somatic mutations. The combination of different layers of gene regulatory information such as, genomic, transcriptomic and epigenomic data will help to identify potential methylation or expression quantitative trait loci (meQTLs and eQTLs), respectively. These features influence methylation or expression across extended genomic regions and may underlie direct SNP associations or gene-environment interactions [26].

Our observations extend the findings obtained by the group of Steiger et al, which linked the glutamate and serotonin systems to AN [28]. Moreover, as other authors have stated, these findings expand our knowledge that the same genetic factors may influence both BMI, and body weight, as well as extreme weight dysregulation like the one occurring in AN [38]. The main strength of our work was that genetic noise was removed and other sources of confounding were also reduced, by analyzing identical twin pairs discordant for AN [16].

### Limitations

The primary strength of this study is that it extends previous investigations by using a twin sample discordant for AN. Nevertheless, it must be considered that this investigation was implemented on DNA samples extracted from peripheral blood rather than from the brain. When the object of study is a body tissue defiant to collect, such as brain tissue, the common strategy is to proceed with more accessible peripheral tissues, such as whole blood. This is under the premise that the variation identified in these ‘proxy’ tissues potentially mirrors the one in the disease-relevant tissue [13]. Moreover, we did not control twin chorionicity. Twins can be sub-classified depending on whether they shared the same placenta or not (monochorionic or dichorionic, respectively). Chorionicity is considered to be influential in epigenetic status [11]. Furthermore, we used a small sample size. However, the novelty, utility, and difficulty to use a twin sample discordant for the disease must be recognised. The present study does not provide evidence of the durability of methylome changes and if they are the cause or a consequence. The performance of future longitudinal epigenetic studies, including recovered patients, may shed some light regarding the causality relationship between our findings and the disease.

To conclude, our discoveries are relevant to healthcare applications. As, currently, AN diagnosis is stablished on the basis of purely clinical criteria, these findings could pave the way for more accurate criteria based on specific epigenetic marks associated with the disease. Moreover, actual treatment strategies are based on targeting symptoms and non-pharmacological intervention has proved to be successful in AN. Improving our knowledge about the physiological bases of AN, which has the highest mortality among the psychiatric conditions [1], is essential to implement adequate diagnosis, prevention, early intervention and treatment strategies. However, to this point, the functional consequences of an altered DNA methylation at the presented CpG sites remain elusive, and future studies have to address their direct association to gene expression and disease-related phenotype changes [16].

## Supplementary information

Supplementary Figure legends

Supplementary Table 1

Supplementary Figure 1

Supplementary Figure 2

Supplementary Figure 3

Supplementary Figure 4

Supplementary Figure 5
