# Supplementary material for: Genome-wide DNA methylation profiling in anorexia nervosa discordant identical twins
Source: Transl Psychiatry. 2022 Jan 10;12:15. doi: 10.1038/s41398-021-01776-y (PMC8748827; doi:10.1038/s41398-021-01776-y)
Supplement: Supplementary file 1 — Supplementary Figure legends [file 41398_2021_1776_MOESM1_ESM.docx]

**Supplementary figure 1. Exploratory analysis of the twin cohort in 2D**

Representation of the Principal Component Analysis (PCA) on the methylation data decomposed in 2 dimensions. Light brown triangles represent anorexic samples and green circles represent control samples.

**Supplementary figure 2. Exploratory analysis of the twin cohort in 3D**

Representation of the Principal Component Analysis (PCA) on the methylation data colored by twin pairs in three dimensions.

**Supplementary figure 3. Exploratory analysis of the twin cohort in 2D**

Representation of the Principal Component Analysis (PCA) on the methylation data decomposed in 2 dimensions.

**Supplementary figure 4. Exploratory analysis of the unrelated cohort in 2D**

Representation of the Principal Component Analysis (PCA) on the methylation data decomposed in 2 dimensions. Light brown triangles represent anorexic samples and green circles represent control samples.

**Supplementary figure 5. DNA methylation levels for the validated CpGs using beta regression between AN and controls in both cohorts**

Box plots of the validated CpGs by beta regression depicting differences in methylation levels between AN and controls in the twin cohort (red) and in the non-twin cohort (blue).
