# Supplementary material for: Genome-wide DNA methylation profiling in anorexia nervosa discordant identical twins
Source: Transl Psychiatry. 2022 Jan 10;12:15. doi: 10.1038/s41398-021-01776-y (PMC8748827; doi:10.1038/s41398-021-01776-y)
Supplement: Supplementary file 2 — Supplementary Table 1 [file 41398_2021_1776_MOESM2_ESM.docx]

| IlmnID | CHR | MAPINFO | Strand | UCSC_RefGene_Name | UCSC_RefGene_Accession | UCSC_RefGene_Group | UCSC_CpG_Islands_Name | Relation_to_UCSC_CpG_Island |
| --- | --- | --- | --- | --- | --- | --- | --- | --- |
| cg05064002 | 19 | 17858612 | F | FCHO1;FCHO1;FCHO1;FCHO1 | NM_001161357;NM_001161358;NM_001161357;NM_001161358 | 5'UTR;1stExon;1stExon;5'UTR | chr19:17858371-17858891 | Island |
| cg13194867 | 14 | 106344321 | R | NA | NA | NA | NA | NA |
| cg07545846 | 11 | 133940520 | F | JAM3 | NM_032801 | Body | chr11:133938850-133939681 | S_Shore |
| cg03031124 | 9 | 131516466 | R | ZER1 | NM_006336 | Body | NA | NA |
| cg24842967 | 1 | 156084750 | F | LMNA;LMNA;LMNA | NM_170707;NM_005572;NM_170708 | 1stExon;1stExon;1stExon | chr1:156084486-156085097 | Island |
| cg08623154 | 1 | 154192647 | F | UBAP2L;C1orf43;C1orf43;UBAP2L;C1orf43 | NM_014847;NM_138740;NM_015449;NM_001127320;NM_001098616 | TSS1500;Body;Body;TSS200;Body | chr1:154192843-154193852 | N_Shore |
| cg02902423 | 4 | 6335372 | R | PPP2R2C;PPP2R2C;PPP2R2C;PPP2R2C;PPP2R2C | NM_181876;NM_001206996;NM_020416;NM_001206995;NM_001206994 | Body;Body;Body;Body;Body | NA | NA |
| cg24792671 | 11 | 70944494 | R | NA | NA | NA | NA | NA |
| cg26633897 | 3 | 26159018 | F | NA | NA | NA | NA | NA |
| cg11540979 | 6 | 158438351 | R | SYNJ2;SYNJ2 | NM_001178088;NM_003898 | 5'UTR;Body | NA | NA |
| cg01682455 | 11 | 45672536 | R | CHST1 | NM_003654 | 5'UTR | chr11:45671197-45672412 | S_Shore |
| cg24533202 | 12 | 49581917 | F | TUBA1A;TUBA1A;TUBA1A | NM_001270399;NM_006009;NM_001270400 | Body;Body;5'UTR | chr12:49582096-49582881 | N_Shore |

Supplementary table 1. Illumina features
