# Supplementary figures and images for: Genome-wide DNA methylation profiling in anorexia nervosa discordant identical twins
Source: Transl Psychiatry. 2022 Jan 10;12:15. doi: 10.1038/s41398-021-01776-y (PMC8748827; doi:10.1038/s41398-021-01776-y)

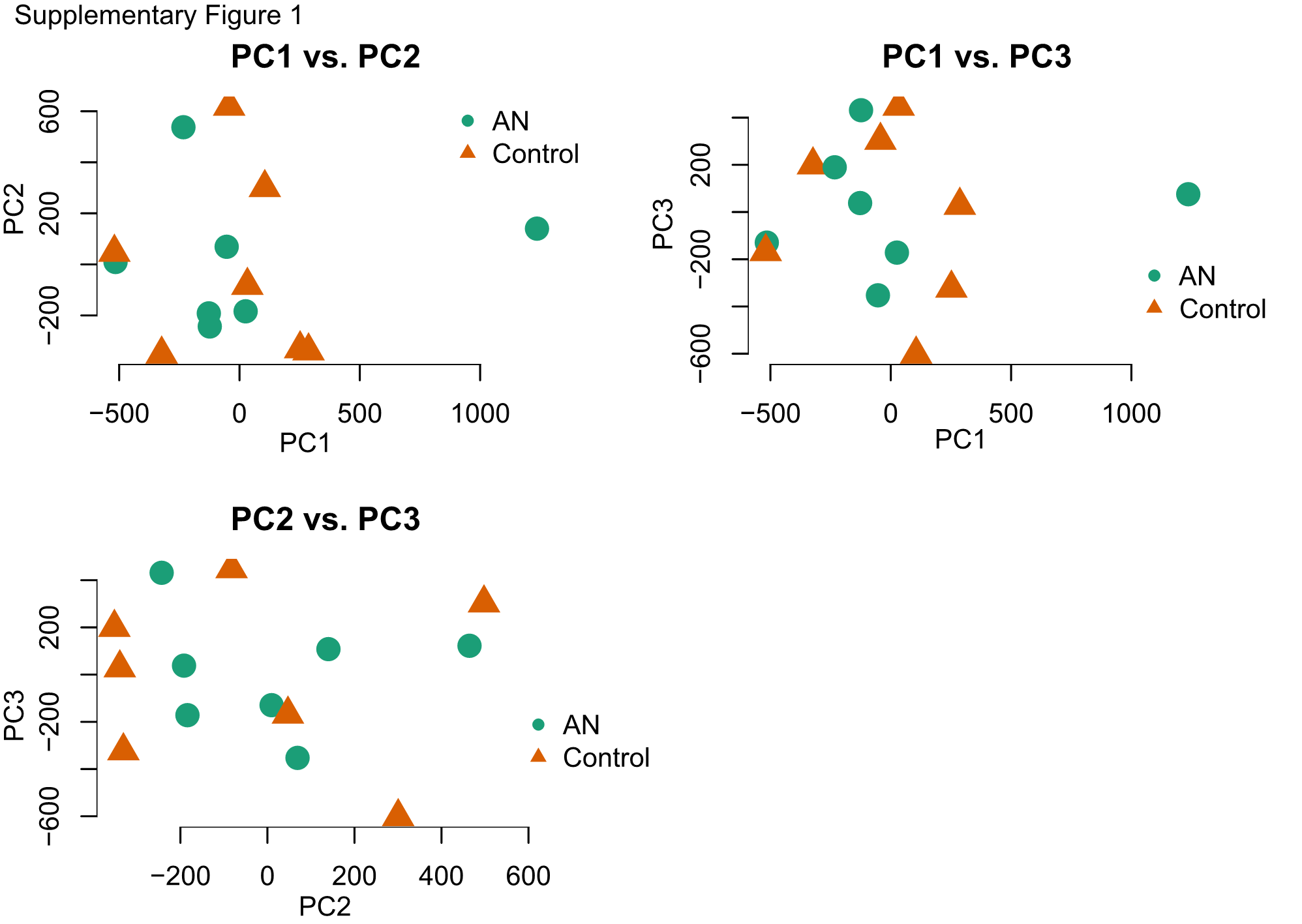

Supplement: Supplementary file 3 — Supplementary Figure 1 [file 41398_2021_1776_MOESM3_ESM.tif]

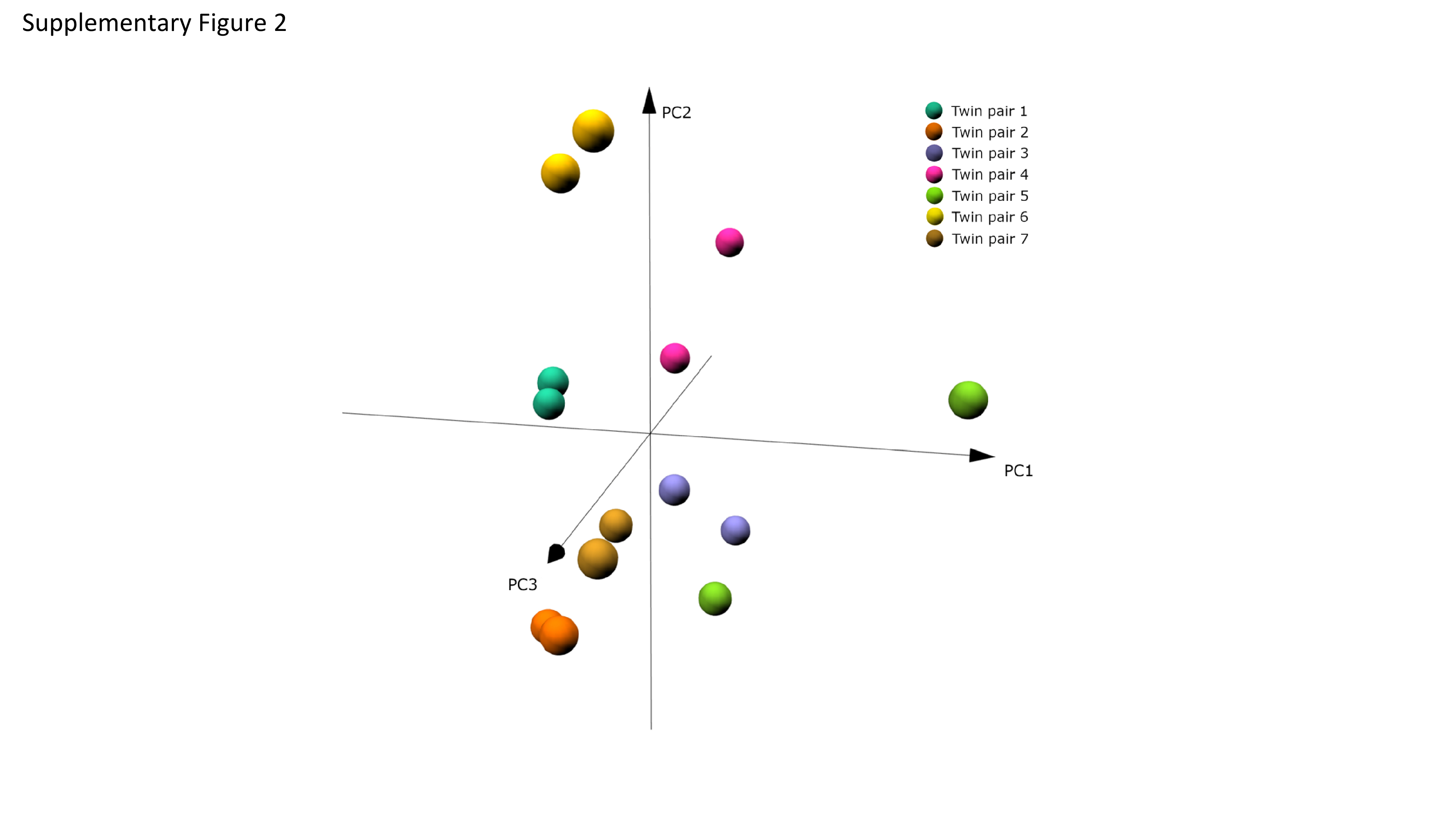

Supplement: Supplementary file 4 — Supplementary Figure 2 [file 41398_2021_1776_MOESM4_ESM.tif]

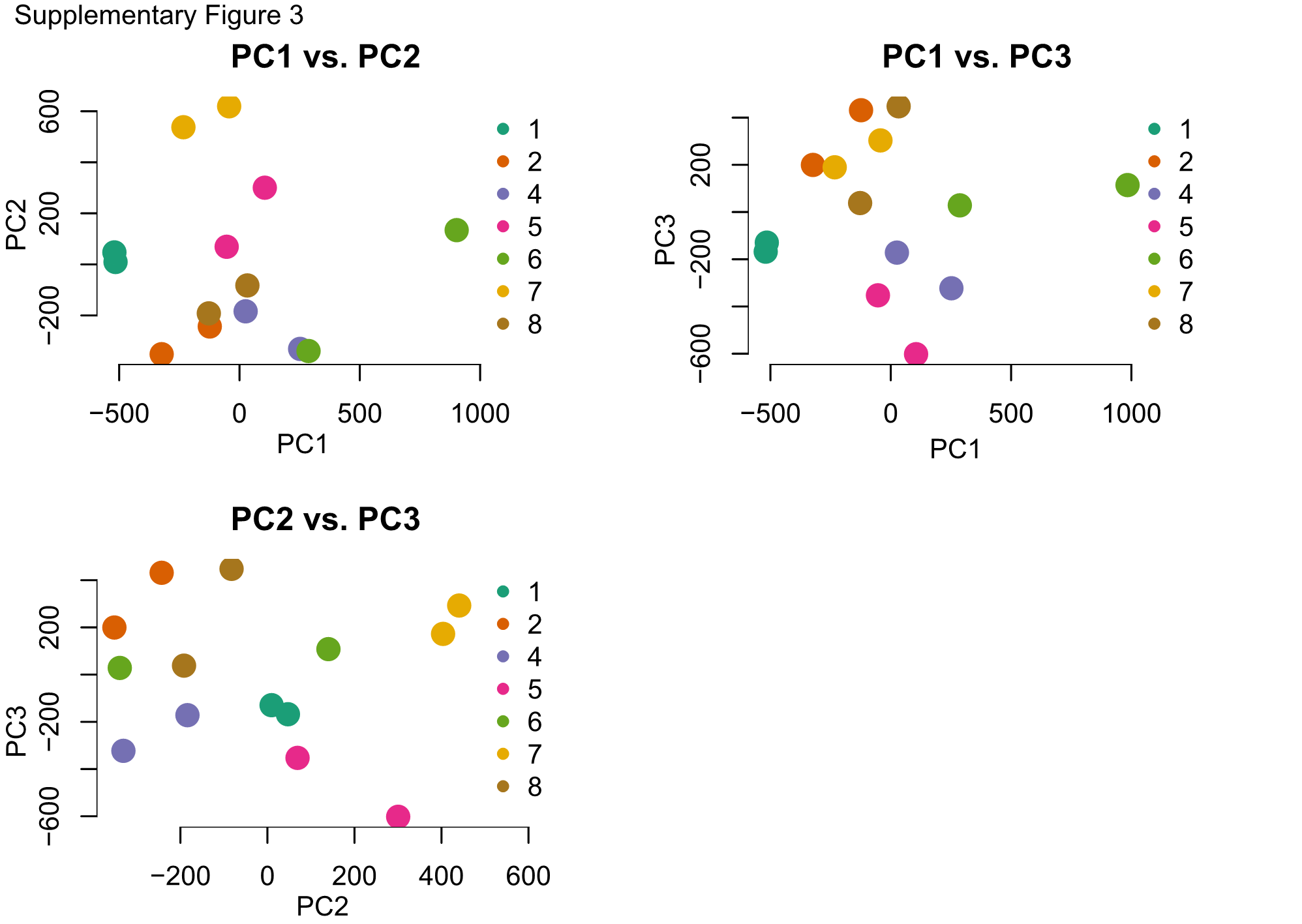

Supplement: Supplementary file 5 — Supplementary Figure 3 [file 41398_2021_1776_MOESM5_ESM.tif]

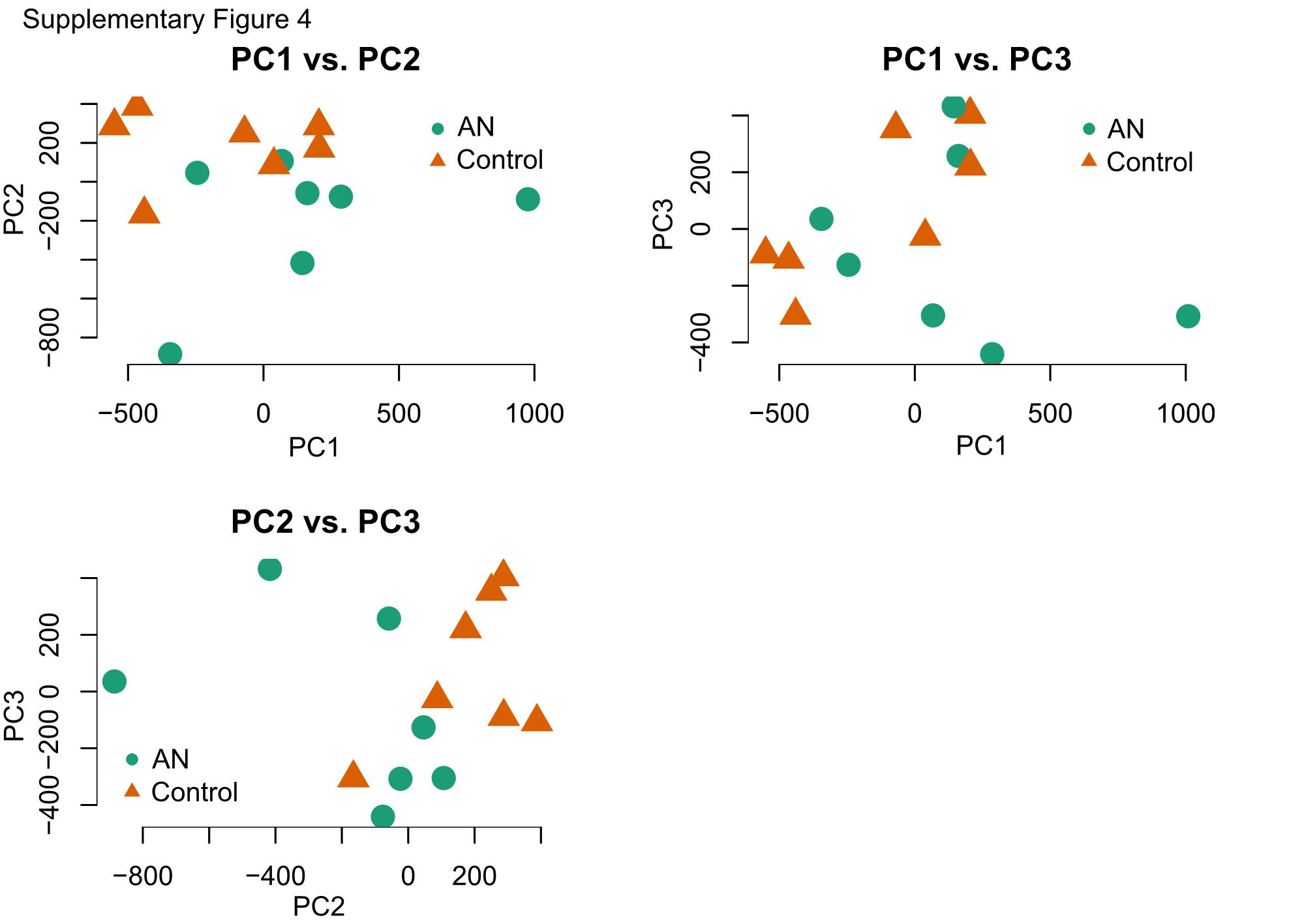

Supplement: Supplementary file 6 — Supplementary Figure 4 [file 41398_2021_1776_MOESM6_ESM.tif]

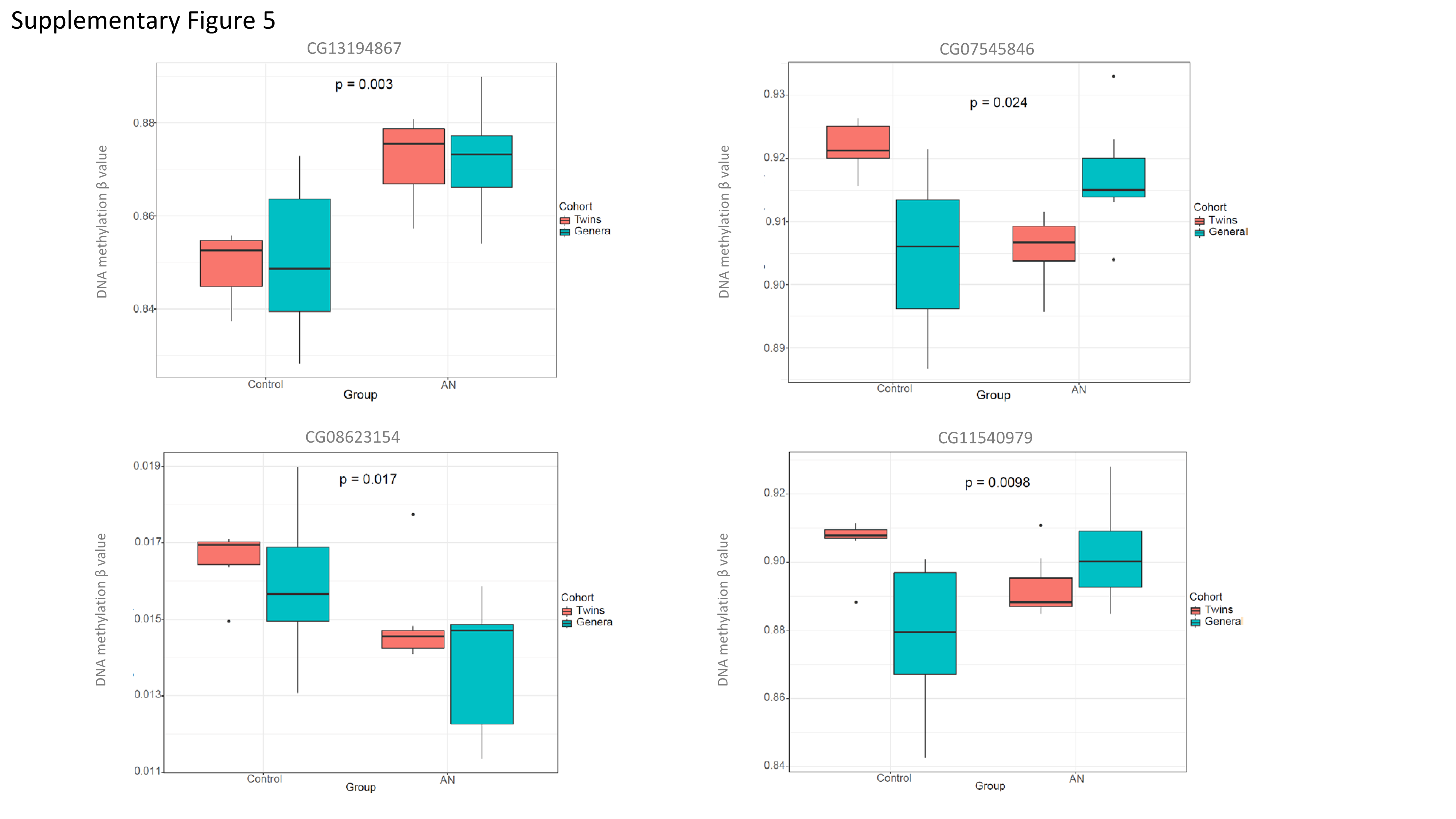

Supplement: Supplementary file 7 — Supplementary Figure 5 [file 41398_2021_1776_MOESM7_ESM.tif]
